# Identification of CTX-M Type ESBL *E. coli* from Sheep and Their Abattoir Environment Using Whole-Genome Sequencing

**DOI:** 10.3390/pathogens10111480

**Published:** 2021-11-14

**Authors:** Nigatu Aklilu Atlaw, Shivaramu Keelara, Maria Correa, Derek Foster, Wondwossen Gebreyes, Awa Aidara-Kane, Lyndy Harden, Siddhartha Thakur, Paula J. Fedorka Cray

**Affiliations:** 1 Department of Population Health and Pathobiology, College of Veterinary Medicine, North Carolina State University, Raleigh, NC 27607, USA; naatlaw@ncsu.edu (N.A.A.); skeelar@ncsu.edu (S.K.); correa@ncsu.edu (M.C.); dmfoster@ncsu.edu (D.F.); lbharden@ncsu.edu (L.H.); sthakur@ncsu.edu (S.T.); 2 Department of Veterinary Preventive Medicine, The Ohio State University, 1920 Coffey Rd., Columbus, OH 43210, USA; gebreyes.1@osu.edu; 3 Department Food Safety and Zoonoses, Foodborne Diseases, World Health Organization, 1202 Geneva, Switzerland; aidarakanea@gmail.com

**Keywords:** abattoir environment, antimicrobial resistance, *E. coli*, ESBL, North Carolina, sheep, whole-genome sequencing

## Abstract

Widespread dissemination of extended-spectrum beta-lactamase (ESBL) *Escherichia coli* (*E. coli*) in animals, retail meats, and patients has been reported worldwide except for limited information on small ruminants. Our study focused on the genotypic characterization of ESBL *E. coli* from healthy sheep and their abattoir environment in North Carolina, USA. A total of 113 ESBL *E. coli* isolates from sheep (n = 65) and their abattoir environment (n = 48) were subjected to whole-genome sequencing (WGS). Bioinformatics tools were used to analyze the WGS data. Multiple CTX-M-type beta-lactamase genes were detected, namely *bla*_CTX-M-1_, *bla*_CTX-M-14_, *bla*_CTX-M-15_, *bla*_CTX-M-27_, *bla*_CTX-M-32_, *bla*_CTX-M-55_, and *bla*_CTX-M-65_. Other beta-lactamase genes detected included *bla*_CMY-2_, *bla*_TEM-1A/B/C_, and *bla*_CARB-2_. In addition, antimicrobial resistance (AMR) genes and/or point mutations that confer resistance to quinolones, aminoglycosides, phenicols, tetracyclines, macrolides, lincosamides, and folate-pathway antagonists were identified. The majority of the detected plasmids were shared between isolates from sheep and the abattoir environment. Sequence types were more clustered around seasonal sampling but dispersed across sample types. In conclusion, our study reported wide dissemination of ESBL *E. coli* in sheep and the abattoir environment and associated AMR genes, point mutations, and plasmids. This is the first comprehensive AMR and WGS report on ESBL *E. coli* from sheep and abattoir environments in the United States.

## 1. Introduction

Extended-spectrum beta-lactamase (ESBL)-producing *Enterobacteriaceae* are a serious public health threat and are increasing worldwide, including in the U.S. [1,2]. *E. coli* are commonly associated with gastro-intestinal, bloodstream, and urinary tract infections [2]. In addition, *E. coli* serves as a reservoir of transferrable antimicrobial resistance (AMR) genes, which can be passed to pathogenic organisms such as *Salmonella* spp. [3,4]. Other ESBL types such as SHV and TEM occurred prior to the emergence of CTX-M type ESBLs; however, CTX-M ESBLs became the leading type in clinical isolates in the early 2000s in the U.S. [5,6]. Later, community dissemination of CTX-M type ESBL *E. coli*, primarily due to *bla*_CTXM-15_ and *bla*_CTXM-14_, was reported among patients in the U.S. [7]. Additionally, CTX-M type ESBLs of food animal origin were first reported in fecal *E. coli* from sick and healthy dairy cattle in Ohio [8]. Nowadays, there are increasing reports of the dissemination of ESBL-producing *E. coli* in food animals, retail meat products, companion animals, and the environment in the U.S. and internationally, which in turn may increase public health risk [9,10,11,12,13,14,15]. 

Dissemination of ESBL *E. coli* in livestock farm-related environments such as soil, water, manure, air, dust, feed, etc., have recently been reviewed [16]. Although beta-lactamase genes including *bla*_CTX-M-1_, *bla*_CTX-M-2_, *bla*_CTX-M-3_, *bla*_CTX-M-8_, *bla*_CTX-M-14_ and *bla*_CTX-M-15_, *bla*_SHV_, *bla*_TEM,_ and *bla*_CMY-2_ were detected in feces of sheep and retail lamb in other parts of the world [10,17,18,19,20], there is no report available on AMR determinants of ESBL *E. coli* in small ruminants in the U.S. Therefore, to fill this gap in information, we conducted a study to detect and characterize AMR determinants using WGS in ESBL *E. coli* recovered from sheep and their abattoir environment in North Carolina.

## 2. Results

### 2.1. AMR Genes and AMR-Associated Point Mutations Detected in ESBL E. coli

**Figure 1:**
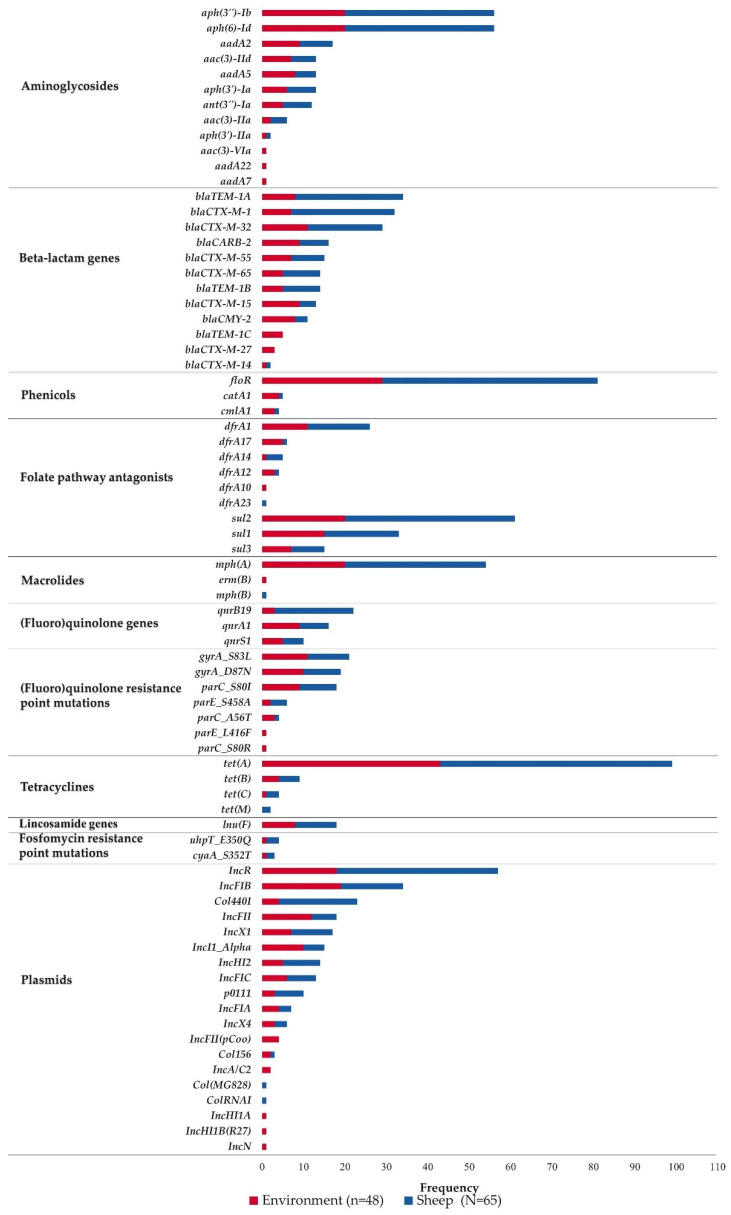
Frequency (%) of AMR determinants detected in ESBL *E. coli* isolates (n = 113) among sample sources.

Molecular characterization of AMR determinants (AMR genes, plasmids, and associated point mutations) of ESBL *E. coli* from sheep and their abattoir environment was conducted using whole-genome sequencing (WGS) data. A total of 113 ESBL *E. coli* isolates from sheep (n = 65) and their abattoir environment samples (n = 48) were included in this study, and results for antimicrobial susceptibility testing against a panel of 14 antimicrobials were obtained. The genotypic tests were 86% (1361/1582) concordant with the phenotypic tests for all tested ESBL *E. coli* isolates (Table 1). The results from 25 phenotypically resistant isolates did not demonstrate a mechanism of resistance, and a total of 196 tests of susceptible isolates carried AMR genes but were not resistant to the specific antimicrobial phenotypically (Table 1). Phenotypic AMR profiles along with the list of detected AMR genes and associated point mutations are shown in Table S1. These ESBL *E. coli* isolates carried a total of 47 different types of AMR genes that confer resistance to at least 10 classes of antimicrobials, 9 different types of AMR-associated point mutations, and 19 different plasmid types (Figure 1 and Table S2). Almost all isolates (98.2%, 111/113) were resistant to at least three classes of antimicrobials, defined as multidrug-resistant (MDR) (Table S1).

Beta-lactamase genes: A total of 22 genotypic profiles of beta-lactamase resistance-conferring genes were detected, including individual or combinations of CTX-M, CARB, TEM, and AmpC type beta-lactamase genes (Table 2). About 96% (108/113) of the ESBL *E. coli* isolates carried CTX-M-type ESBL encoding genes. Phenotypically, all study isolates were resistant to Ceftriaxone (MIC ≥ 4 µg/mL), and Ampicillin (MIC ≥ 32 µg/mL) and all except one isolate were resistant to Ceftiofur (MIC ≥ 8 µg/mL). We report 7 unique CTX-M-type ESBL genes from the 113 ESBL *E. coli* from sheep and their abattoir environment, namely *bla*_CTX-M-1_ (28.3%, 32/113), *bla*_CTX-M-14_ (1.8%, 2/113), *bla*_CTX-M-15_ (11.5%, 13/113), *bla*_CTX-M-27_ (2.7%, 3/113), *bla*_CTX-M-32_ (25.7%, 29/113), *bla*_CTX-M-55_ (13.3%, 15/113) and *bla*_CTX-M-65_ (12.4%, 14/113) (Figure 1 and Table S2). Other beta-lactamase genes detected were *bla*_TEM-1_ (46.9%, 53/113), *bla*_CARB-2_ (14.2%, 16/113) and the AmpC beta-lactamase gene, *bla*_CMY-2_ (9.7%, 11/113) (Figure 1 and Table S2). Three types of *bla*_TEM-1_ genes were detected: *bla*_TEM-1A_ (30.1%, 34/113), *bla*_TEM-1B_ (12.4%, 14/113) and *bla*_TEM-1C_ (4.4%, 5/113). None of the CTX-M type ESBL genes were found in five isolates (Table 2). Of these, four carried a combination of *bla*_CMY-2_ and *bla*_TEM-1C,_ and one carried *bla*_CMY-2_ without additional beta-lactamase genes. 

**Figure 2:**
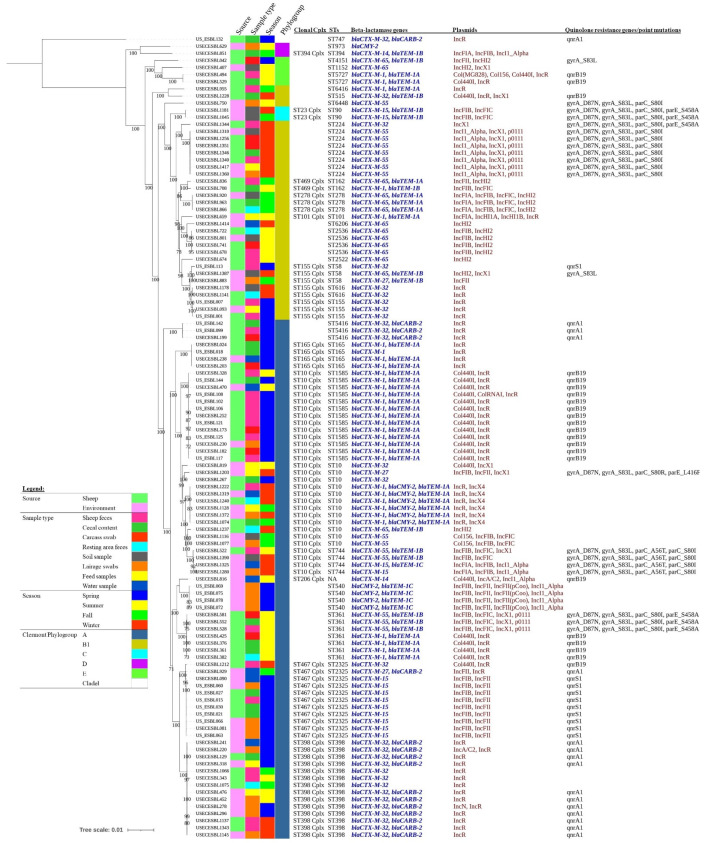
Midpoint rooted phylogenetic tree constructed based on maximum-likelihood of core-genome alignment from 113 ESBL *E. coli*. The alignment was built using Roary version 3.13.0. The ML phylogenetic tree was made using RaxMLGUI2.0 with the best fitting model GTR + I + G. Visualization and annotation was carried out through iTOL version 6.3 (https://itol.embl.de/itol.cgi; accessed on 19 July 2021). Bootstrap values between 70% and 100% are shown. The total number of core genes was 3049 and the total number of alignment sites was 2988599. Cplx = complex; STs = Sequence types.

The five most frequent beta-lactam genes found together or alone were *bla*_CTX-M-1_ and *bla*_TEM-1A_ (21.2%, 24/113), *bla*_CTX-M-32_ and *bla*_CARB-2_ (13.3%, 15/113), *bla*_CTX-M-32_ (11.5%, 13/113), *bla*_CTX-M-15_ (8.8%, 10/113) and *bla*_CTX-M-55_ (8.8%, 10/113) (Table 2). The remaining mechanisms of beta-lactam resistance are presented in Table 2. All beta-lactamase genes reported had 100% length coverage and 100% identity to previously published beta-lactamase genes. Seven out of 11 isolates that carried the *bla*_CMY-2_ gene were resistant to Cefoxitin and Amoxicillin/Clavulanic acid (Figure 2). The rest of the four isolates carried *bla*_CMY-2_ with *bla*_TEM-1C_; however, they were susceptible to these antimicrobials_._ All Amoxicillin/Clavulanic acid-resistant ESBL *E. coli* isolates (MIC ≥ 32/16 µg/mL) were also resistant to Cefoxitin (MIC ≥ 32) (n = 9). Of these, the majority (n = 6) carried a combination of *bla*_CTX-M-1_, *bla*_CMY-2_ and *bla*_TEM-1A_, while others carried *bla*_CTX-M-1_ and *bla*_TEM-1A_ (n = 1), *bla*_CTX-M-32_ and *bla*_CARB-2_ (n = 1) or *bla*_CMY-2_ (n = 1) alone. The isolate with *bla*_CMY-2_ alone as the beta-lactamase gene was susceptible to Ceftiofur (MIC = 4µg/mL) and had the lowest MIC value for Ceftriaxone (8 µg/mL) (Table S1 and Figure 2). The list of and percent detection of known AMR genes, including other classes of antimicrobials, AMR-associated point mutations, and plasmids are shown in Table S2.

Aminoglycosides: Phenotypic aminoglycoside-resistant ESBL *E. coli* (n = 87) isolates, as determined by resistance to Gentamicin (MIC ≥ 16 µg/mL) and/or Streptomycin (MIC ≥ 32 µg/mL), carried at least one gene known to confer this resistance, except in one isolate where the resistance mechanism was not identified (Table 1). Aminoglycoside-resistant isolates carried a total of 23 different genotypic profiles; the top three profiles were *aph(3’’)-Ib* (or *strA*) and *aph(6)-Id* (or *StrB*) (31.0%, 35/113), *aadA2* alone (12.4%, 14/113), and *aadA5, aph(3”)-Ib* and *aph(6)-Id* (8.0%, 9/113) (Table S3).

Macrolides: Most (40/45) of the Azithromycin (a macrolide)-resistant isolates (MIC ≥ 32 µg/mL) carried *mph(A)*; however, a known macrolide resistance mechanism was not detected in five isolates (Table 1). One Azithromycin-resistant isolate carried an additional mechanism, *erm(B)* (Table S3). However, several ESBL *E. coli* isolates that carried either *mph(A)* or *mph(B)* (n = 15) were phenotypically susceptible to Azithromycin (Table 1).

Phenicols: Chloramphenicol-resistant ESBL *E. coli* isolates (n = 87, MIC ≥ 32 µg/mL) carried either *floR* (n = 65.5%, 74/113) or *catA1* (1.8%, 2/113) or combinations of *floR* and *cmlA1* (3.5%, 4/113) or *floR* and *catA1* (2.7%, 3/113) (Table S3). Genes that conferred phenicol resistance were not detected in four phenotypically Chloramphenicol-resistant ESBL *E. coli* isolates (Table 1).

Quinolones/Fluoroquinolones: All ESBL *E. coli* isolates phenotypically resistant to Ciprofloxacin, a fluoroquinolone (n = 19, MIC ≥ 4 µg/mL), carried at least three substitutions: two substitutions at quinolone resistance-determining regions (QRDR) of the gene for DNA gyrase (*gyrA*_D87N and *gyrA*_S83L) and all except one had additional substitution at topoisomerase IV (*parC*_S80I) and the remaining one isolate at *parC*_S80R). Nearly half of these isolates (11/19) carried a fourth substitution at topoisomerase IV (either *parC*_A56T (n = 4), *parE*_S458A (n = 6) or *parE*_L416F (n = 1)) (Table S1 and Table S3). Two isolates (USECESBL042 and 1387) with a single substitution at the gene for DNA gyrase, *gyrA*_S83L, were resistant to Nalidixic acid but not resistant to Ciprofloxacin (Table S1). ESBL *E. coli* isolates carried plasmid-mediated quinolone resistance (PMQR) genes, namely *qnrA1* (14.2%, 16/113), *qnrB19* (19.5%, 22/113), and *qnrS1* (8.8%, 10/113), but none of these isolates had quinolone resistance-associated point mutations (Table S1 and Figure 2). Among these isolates with PMQR, only three isolates which harbored *qnrB19* were resistant to Nalidixic acid; the rest of the isolates were not resistant to both Nalidixic acid and Ciprofloxacin. Two Nalidixic acid-resistant isolates did not carry any known quinolone resistance determinants (Table S1 and Figure 2).

Folate pathway antagonists: Among all tested isolates, nearly 40% (45/113) carried *sul2* and 22.1% (25/113) carried *sul1* and *dfrA1* (Table S3). The remaining isolates exhibited 12 different genotypic profiles of resistance against folate-pathway antagonists. Among isolates resistant to folate-pathway antagonists (93/113), all Trimethoprim/Sulfamethoxazole (MIC ≥ 4/76 µg/mL)-resistant isolates (40/113) were also resistant to Sulfisoxazole (MIC ≥ 512 µg/mL) (Table 1 and Table S1). *Sul*-type genes were not detected in two Sulfisoxazole-resistant isolates and an isolate susceptible to Sulfisoxazole and Sulfamethoxazole-Trimethoprim carried both *sul1* and *dfrA1* genes. Similarly, *dfrA-*type genes were not detected in two Sulfamethoxazole-Trimethoprim-resistant isolates. In contrast, *dfrA1* was detected in four isolates that were phenotypically categorized as sensitive to Sulfamethoxazole-Trimethoprim (Table S1).

Tetracyclines: From a total of 110 Tetracycline-resistant (MIC ≥ 16) ESBL *E. coli*, 103 (93.6%) carried at least one gene known to confer Tetracycline resistance (Table 1). These isolates carried either *tet(A)* (78.8%, 89/113), *tet(B)* (3.5%, 4/113), *tet(A)* and *tet(B)* (4.4%, 5/113), *tet(A)* and *tet(C)* (3.5%, 4/113) or *tet(A)* and *tet(M)* (0.9%, 1/113) (Table S3). One isolate that carried *tet(M)* was phenotypically sensitive to Tetracycline. Seven Tetracycline-resistant ESBL *E. coli* isolates did not carry any of the above Tetracycline-conferring genes (Table 1 and Table S1).

Lincosamides and Fosfomycin: Lincosamide nucleotidyltransferase coding gene, *Inu(F)*, which confers resistance to lincomycin was detected in some ESBL isolates (15.9%, 18/113) (Figure 1, Table S2 and Table S1). In addition, Fosfomycin resistance-conferring regulatory gene mutations in either *cyaA*_S352T (n = 2), *uhpT_*E350Q (n = 3), or both (n = 1) were detected in ESBL *E. coli* isolates in this study (Table S1 and Table S3). However, the ESBL *E. coli* isolates were not evaluated for phenotypic susceptibility to Lincosamides and Fosfomycin.

### 2.2. AMR Determinants among Sample Types and Seasons

Most of the AMR gene types (37/47) and point mutation types (7/9) detected in this study were carried by ESBL *E. coli* isolates from both sheep and environment sources (Figure 1 and Table S2). The exception to this included *bla*_CTXM-27_, *bla*_TEM-1C_, *aac(3)-VIa*, *aadA22*, *aadA7*, *dfrA10*, *ermB*, and two substitutions at QRDR (*parC*_S80R and *parE*_L416F). These genes and point mutations were not detected in isolates from sheep samples. On the other hand, *dfrA23*, *mphB,* and *tet(M)* were not detected in isolates from the environmental samples. Carriage of AMR determinants differed between seasons, and only about 44.5% (21/47) AMR gene types and 14.3% (1/7) of the types of substitutions at QRDR were detected in all seasons of the study. Of these, 12 types of AMR genes (*bla*_CTXM-1_, *bla*_CTXM-32_, *bla*_TEM-1A_, *aph(3”)-Ib*, *aph(6)-Id*, *floR, mphA*, *dfrA1*, *sul1*, *sul2*, *tet(A)* and *tet(B)*) were detected in two or more isolates per season (Figure 1 and Table S2). Among beta-lactamase genes, all ESBL *E. coli* isolates from carcass swabs (n = 10) carried CTX-M type ESBL genes including *bla*_CTX-M-1_ (n = 4), *bla*_CTX-M-55_ (n = 3), *bla*_CTX-M-65_ (n = 2) and *bla*_CTX-M-32_ (n = 1) (Table 2). These isolates were recovered in spring (n = 5), summer (n = 3), and winter (n = 2) seasons (Figure 2).

### 2.3. Characterization of Plasmids in ESBL E. coli from Sheep and Abattoir Environment

Plasmids (19 different types) were detected in 96% (109/113) of the ESBL *E. coli* isolates (Figure 2). The most common types of plasmids detected were IncR (50.4%, 57/113), IncFIB (30.1%, 34/113), and Col440I (20.4%, 23/113) (Figure 1 and Table S2). The majority of the isolates carried more than one plasmid. The top five plasmid profiles(s) detected in ESBL *E. coli* isolates were IncR alone (23.0%, 26/113), Col440I and IncR (15.9%, 18/113), IncFIB and IncFII (8.0%, 9/113), IncI1_Alpha, IncX1 and p0111 (6.2%, 7/113), and IncR and IncX4 (5.3%, 6/113) (data not shown). Isolates shared all plasmid types from both sheep and environment sources, except that IncA/C, IncFIIpCoo, IncHI1A, IncHI1B, and IncN were detected only in isolates from the abattoir environment, and Col(MG828) and ColRNAI were detected only in isolates from sheep samples. Carriage of plasmids varied between seasons, and only four types of plasmids (IncFIB, IncR IncHI2, and IncI1-Alpha) were detected in all seasons of the study (Figure 1 and Table S2).

### 2.4. Sequence Types and Phylogenetic Analysis of ESBL E. coli Isolates

**Figure 3:**
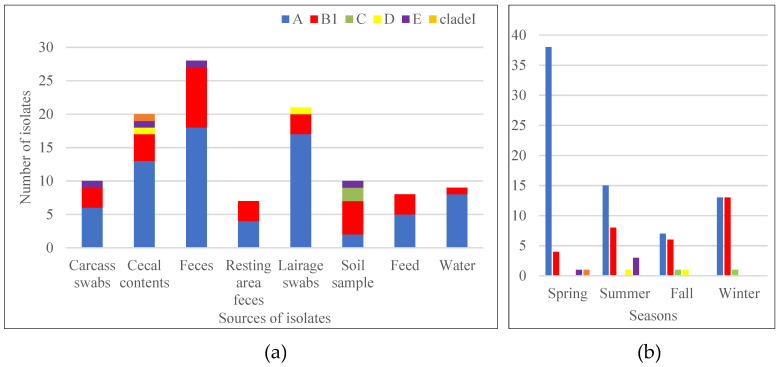
Type and number of phylogroups of ESBL *E. coli* recovered from the different sample types (**a**) and among the four seasons (**b**). Phylogroups were determined using Clermont Typing. Phylogroups are indicated with different colors: blue for phylogroup A, red for phylogroup B1, light green for phylogroup C, yellow for phylogroup D, purple for phylogroup E, and orange for cladeI. (**a**) indicates that phylogroup A and B1 were commonly found in all sample types, phylogroup C was found only in soil samples, phylogroup D was found in cecal content and lairage swab, phylogroup E was found in cecal content, sheep feces, carcass swab and soil samples and CladeI was found in cecal content. (**b**) indicates number of the different phylogroups recovered in the four seasons. Phylogroups A and B1 were found in all seasons. Phylogroups C, D, and E were each detected in two seasons. CladeI was found only in spring season.

ClermonTyping of 113 ESBL *E. coli* isolates showed that most of the ESBL *E. coli* isolates belonged to phylogroup A (73/113, 64.6%) and phylogroup B1 (31/113, 27.4%). The remaining nine isolates were assigned to phylogroup C and D (two isolates each), phylogroup E (four isolates), and CladeI (one isolate). Distributions of phylogroups of ESBL *E. coli* isolates among the different sample types and seasons are shown in Figure 3.

A total of 38 different serotypes were detected, with the most predominant ones being O8:H20 (12.4%), -:H32 (11.5%), O9:H30 (9.7%), O10:H25 (8.0%) and -:H23 (6.2%). Twelve out of the 38 different serotypes were detected both in ESBL *E. coli* from sheep and the abattoir environment and included O10:H25, O100:H32, O178:H7, O32:H10, O8:H20, O8:H9, O9:H30, -:H23, -:H26, -:H28, -:H32 and -:H34 (Table S1).

Twenty-nine different sequence types (STs) were detected from all tested ESBL *E. coli* isolates, and 12 of the STs were detected in isolates from both sheep and abattoir environment samples. The top ten common sequence types, accounting for 72% of the isolates, were ST398 (14/113), ST1585 (13/113), ST10 (12/113), ST2325 (11/113), ST224 (8/113), ST361 (7/113) and ST165, ST540, ST744 and ST2536 (4/113 each). ST for one isolate (Isolate ID: USECESBL816, SRR11347457) was not identified by the MLST database. Twelve out of the 29 STs (ST398, ST585, ST10, ST2325, ST224, ST165, ST744, ST2536, ST58, ST155, ST278, and ST616) were detected in ESBL *E. coli* isolates from both sheep and the abattoir environment (81/113, 71.7%). Fifteen unique STs of ESBL *E. coli* were detected in sheep feces, followed by cecal content (14 STs), abattoir resting area feces (12 STs), and lairage swab (10 STs), and the least diversified were isolates from feed samples (5 STs) (Figure 2). ST398 and ST10 were detected in all seasons of the study duration, while two STs (ST58 and ST2325) were detected in three seasons (fall, spring, and winter), and the majority (21/29) of the unique STs were detected only in a season. However, 14 unique STs of ESBL *E. coli* were detected in summer, followed by spring (11 STs), winter (10 STs), and fall (9 STs) (Figure 2). The core-genome phylogenetic analyses of the ESBL *E. coli* isolates revealed that sequence types of isolates were more clustered based on season than based on source or type of samples (Figure 2).

## 3. Discussion

To our knowledge, this is the first report of molecular characterization of AMR determinants in ESBL *E. coli* from sheep and their abattoir environment in the U.S. The isolates were obtained from a year-round serial cross-sectional study between March 2019 and February 2020 in North Carolina. In this study, 95.6% (108/113) of the phenotypically confirmed ESBL *E. coli* carried CTX-M-type beta-lactamase genes as mechanisms of ESBL production. The most predominant beta-lactamase genes detected in our study were *bla*_CTX-M-1_ and *bla*_CTX-M-32_ followed by *bla*_CTX-M-55_, *bla*_CTX-M-65_, *bla*_CTX-M-15_, *bla*_CTX-M-27,_ and *bla*_CTX-M-14_. In the U.S., *bla*_CTX-M-1_ was reported as the predominant CTX-M-type ESBL gene in *E. coli* recovered from environmental samples from dairy farms, livestock auction markets, and equine facilities [21]. However, *bla*_CTX-M-15_ is the predominant and widely disseminated ESBL gene carried by ESBL *E. coli* from dairy cattle farms in other locations and human urinary tract infections in the U.S. [7,21,22,23]. In cattle and humans, *bla*_CTX-M-27_ and *bla*_CTX-M-14_ were also commonly reported in these studies. We detected *bla*_CTX-M-15_ in ESBL *E. coli* from 13 isolates recovered from cecal contents, sheep feces, lairage swabs, soil sample, and water, while *bla*_CTX-M-14_ and *bla*_CTX-M-27_ were less frequent and detected in only two and three isolates, respectively. Six ESBL *E. coli* isolates (O100:H32, ST10) recovered from both sheep and the abattoir environment in our study carried a combination of three beta-lactamase genes: *bla*_CTX-M-1_ (broad-spectrum ESBL gene), *bla*_TEM-1A_ (narrow spectrum), and *bla*_CMY-2_ (AmpC type beta-lactamase gene). Such ESBL *E.coli* isolates were previously termed as mixed ESBL/AmpC phenotype [24]. CTX-M-type and SHV-type ESBL genes were found to coexist in ESBL *E. coli* from sheep meat in China [11]. *bla*_SHV_, *bla*_OXA,_ and *aac(6)-Ib-cr* type beta-lactamase genes were not detected from both sources in our study, which may restate the current predominance of CTX-M and TEM-type ESBLs in *E. coli* [25]. A combination of CTX-M and TEM type beta-lactamase genes had been reported in ESBL *E. coli* isolates from sheep in Turkey [19], while Lui et al. (2016) reported up to eight different beta-lactamase genes in ESBL *E. coli* from a dog with severe urinary tract infection in the U.S., including four different CTX-M-types and four other types (TEM, CMY, SHV, and aac(6′)-Ib-cr) of beta-lactamase genes.

In this study, five ESBL *E. coli* isolates carried the AmpC type beta-lactamase gene, *bla*_CMY-2_ with *bla*_TEM-1C_ (n = 4) or alone (n = 1) and did not carry the ESBL gene. The genes known for ESBL production were not detected in these isolates. This observation could be due to other undetected genes or false-positive results in the determination of ESBL status at the screening phase, as previously observed in other studies [26,27]. The other two ESBL producer isolates were resistant to Cefoxitin and Amoxicillin/Clavulanic acid in the absence of *bla*_CMY-2_. These isolates carried ESBL genes *bla*_CTX-M-1_ and *bla*_CTX-M-14_ combined with *bla*_TEM-1A_ and *bla*_CARB-2_, respectively. This discrepancy of phenotypic and genotypic results could be the lack of expression of genes in the genotypically predicted resistant but phenotypically susceptible isolates to infer resistance, as previously noticed [28].

This is the first report of multiple beta-lactamase genes in ESBL *E. coli* from sheep in the United States. Wide dissemination of multiple types of beta-lactamase genes was previously reported from cattle and retail meats excluding lamb and goat in the U.S. [8,9,23] and companion animals (dogs and cats) [12]. From the U.S. public health sector, the most commonly reported CTX-M type genes in ESBL *E. coli* were *bla*_CTX-M-15_ and *bla*_CTX-M-14_ [5,7,22,29]. These studies also reported multiple types of beta-lactamase genes in patients with urinary tract and bloodstream infections and pneumonia, including *bla*_CTX-M-3_, *bla*_CTX-M-16,_ *bla*_CTX-M-27,_ *bla*_CTX-M-107,_ *bla*_SHV-2,_ *bla*_SHV-5,_ *bla*_SHV-12,_ *bla*_TEM-1,_ and *bla*_TEM-10_. McGann et al. reported detection of a plasmid-borne colistin resistance gene, mcr-1, *bla*_CTX-M-55,_ and *bla*_CTX-M-15_ from ESBL *E. coli* isolates from urinary tract infection in the U.S. [30]. In a study conducted on ESBL *E. coli* from lamb meat in Brazil, MDR and potentially pathogenic isolates harboring *bla*_CTX-M-2_, *bla*_CTX-M-8_, *bla*_CTX-M-14,_ and *bla*_CTX-M-55_ were recently reported [31]. Hence, our study and others indicate the presence and dissemination of clinically important beta-lactamases in *E. coli* in sheep, their products, and the abattoir environment, and the necessity for routine surveillance of these pathogens.

Moreover, ESBL *E. coli* from sheep and the abattoir environment carried AMR genes conferring resistance to Tetracyclines, Sulfonamides, Aminoglycosides, phenicols, Quinolones, Macrolides, Trimethoprim, and Lincosamide. AMR-associated point mutations at *gyrA*, *parC,* and *parE* that confer resistance to fluoroquinolones and at *uhpT* and *cyaA* that confer resistance to Fosfomycin were detected in these pathogens [32]. From all detected AMR genes in our study, ESBL *E. coli* from sheep carried a higher proportion of *bla*_CTX-M-1_, *bla*_TEM-1A_, *floR*, *qnrB19,* and *sul2,* while those from the environment carried a higher proportion of *bla*_CTX-M-15_ and *bla*_TEM-1C_. Our study detected genotypic determinants of AMR in ESBL *E. coli* that were more diversified than in previous reports from cattle and retail meats in the U.S. [9] and sheep in Spain and Portugal [10]. The higher percentage of AMR genes in the sheep in our study could be due to inadequate biosecurity measures, including mixing of animals (sheep, goats, and cattle) from different farms and county fairs, sharing of contaminated feed and water from common sources at the abattoir resting area and prolonged time of duration for interaction, or sharing of AMR bacteria and the associated horizontal gene transfer between them [33]. Although our study did not evaluate these plausible reasons, it was reported that environmental samples from county fairs and livestock auction markets carried a higher level of Cephalosporin and fluoroquinolone-resistant *E. coli* than those from individual facilities for dairy cattle, equine, or companion animals [21]. At the study abattoir, sheep, goats, and cattle were allowed to roam around for a few hours to up to three days before slaughter. The abattoir operates year-round, receiving animals from different sources, which further increases the chance of introducing diversified genotypes of bacteria. We noticed that the abattoir routinely conducted proper cleaning and applied antiseptics on the lairage at the end of each slaughter day. However, the abattoir resting area was muddy and/or dusty, which might allow immediate contamination of the lairage. We detected a higher diversity of AMR genes in the abattoir environment and recovered a higher percentage of *Salmonella* and ESBL *E. coli* in abattoir environmental samples, which supports this observation (data not shown). Another contributing factor could be a large number of animals packed per waiting pens/cubicles as observed during the study. 

From the 19 different types of plasmids detected in our study, about 70% of ESBL *E. coli* isolates carried two or more types. These were primarily incompatibility (Inc type) and colicinogenic (Col type) plasmids. Most plasmids detected in ESBL/AmpC *E. coli* were reported to be plasmid-mediated [10]. From all plasmids detected in this study, IncA/C, IncF, IncI1-Alpha, IncN, and IncH were previously found to be associated with MDR and commensal *E. coli* [34,35]. Combining all types of IncF plasmids (IncFIA, IncFIB, IncFIC, IncFIIpCoo, and IncFII), IncF was detected in more than two-thirds (76/113) of the ESBL *E. coli* isolates, indicating that they were the leading carriers of ESBL genes as previously noted [35]. IncR plasmids were the second abundant (57/113) types of plasmids in our study. IncR plasmid was described to carry genes belonging to many classes of antimicrobials, including beta-lactams and quinolones [35].

Multiple sequence types (n = 29) were found to harbor CTX-M-type ESBL genes in our study. From these, at least eight of the STs, namely, ST10, ST58, ST90, ST162, ST361, ST540, and ST744, were previously reported in ESBL *E. coli* from dairy cows [23] and ST10, ST58, ST398, and ST540 were reported from fluoroquinolone-resistant *E. coli* from retail meats (ground turkey and pork chops) in the U.S. [36]. However, this study did not detect major pandemic lineages such as ST131, ST393, ST69, ST95, and ST73 (Riley, 2014). The carbapenemase gene, *bla*_NDM-1_ was not detected in our study. However, in our research, an isolate from feed belongs to ST101, associated with the New-Delhi metallo-beta-lactamase encoding gene (*bla*_NDM-1_)[37,38]. 

In this study, most of the isolates were phylogroups A (73/113) and B1 (31/113), followed by E (4/113), C (2/113), D (2/113), and CladeI (1/113), and all except phylogroup C were detected in isolates from sheep samples. Phylogroup A was detected at a higher proportion in isolates from all sample types except those from soil samples, where a higher proportion of phylogroup B1 was detected. ESBL *E. coli* isolates from cecal content had the most diversified phylogroups (A, B1, D, E, and CladeI). An abattoir-based study in Portugal indicated that 92.6% (50/54) of *E. coli* recovered from sheep were phylogroup A and B1 [39], the remaining two each from phylogroup B2 and D. However, the proportion of B1 was about twice the proportion of A1 in their study, contrasting the result in our study. Similarly, the predominance of phylogroups A and B1 in *E. coli* was reported in ruminants (cattle and sheep) in Turkey. In addition, they reported phylogroup D both from cattle and sheep but did not report other phylogroups [19]. Phylogroup B2 and D are considered pathogenic [40]. Two isolates in our study were phylogroup D.

Of the 38 different serotypes of ESBL *E. coli* detected in our study, one was O45, which is among the most common serogroups of non-STEC capable of causing disease in humans [41]. Among the identified serotypes, at least seven of them were considered noble serotypes by the EcoH database, including O5:H21, O9:H34, O10:H29, O22, or O32:H9, O24:H32, O31:H15, and O32:H10.

The phylogenetic analyses revealed that most of the unique sequence types tend to cluster around seasons but not around sample type or source of isolates. This may suggest close interaction between animals at the slaughter facility and the abattoir environment, facilitating the sharing of bacteria and AMR genes. Although only ST10 and ST398 were detected across all seasons and ST58 and ST2325 were detected in three seasons, these isolates were clonal, indicating persistence in the environment and animals throughout the year. This could be due to differences in bacterial fitness, previous environmental dissemination, and livestock farms and markets where the animals come from. It was interesting to see that these STs harbored diverse types of beta-lactamase genes. ST10 isolates harbored eight unique types of beta-lactamase genes (five CTX-M-types, AmpC type, and two TEM-types), ST58 and ST2325 harbored three CTX-M types, and the former had one TEM type beta-lactamase gene. However, isolates with ST398 harbored only *bla*_CTX-M-32_ and *bla*_CARB-2_. This might need further investigation. A recent report indicated such fitness differences could be associated with plasmid–host adaptations [42].

Core genome phylogenetic analyses indicated that almost all types of beta-lactamase genes were scattered throughout the phylogenetic tree. Similar STs were detected in isolates recovered from both sheep and the environment. These may further indicate close interaction and mobile genetic transfer of acquired AMR genes between isolates from both sources. For example, six clonal ESBL *E. coli* isolates (O100:H32; ST10-A) that carried a combination of three beta-lactam genes were recovered from six different samples and detected in two seasons (fall and winter).

The study had limitations, as some important demographic information was not accessible such as the history of illnesses and antimicrobial use, geographical source of animals, history of transportation, dietary changes, and husbandry management. The study did not evaluate the possible contribution of cattle and goats at the same facility in the dissemination of ESBL *E. coli* and AMR genes. Additionally, we did not look into the effect of transportation and abattoir environment in acquiring AMR genes and their dissemination to sheep and their products.

In conclusion, this is the first comprehensive report of AMR determinants in ESBL *E. coli* from sheep and their abattoir environment in the U.S. Sheep are a significant reservoir of ESBL *E. coli* and AMR determinants, and this study notably indicated close interaction between ESBL *E. coli* from sheep and their abattoir environment. The abattoir environment might have played a significant role in the persistence and dissemination of these pathogens. We propose routine AMR surveillance of sheep and their products to prevent future public health risks.

## 4. Materials and Methods

### 4.1. Study Design and Bacterial Isolates

From the pool of ESBL *E. coli* isolates recovered during a serial cross-sectional study conducted between March 2019 and February 2020, we selected 113 ESBL *E. coli* isolates for molecular characterization of AMR determinants. The selected isolates were recovered from sheep samples (n = 65) and abattoir environment samples (n = 48). Break down of samples collected and sampling methodology are described in Table S4. Sources of ESBL *E. coli* isolates from sheep were carcass swabs (n = 10), feces (n = 28), cecal contents (n = 20), and abattoir resting area feces (n = 7), and those from the abattoir environment were lairage swabs (n = 21), soil (n = 10), feed (n = 8) and water (n = 9). The abattoir slaughtered sheep, goats, and cattle on a routine basis. These animals were allowed to roam around from a few hours to up to three days and share feed and water from the same troughs. Information on antimicrobial use, husbandry, and demography was not accessible to us. ESBL *E. coli* isolates were selected based on their AMR profile, the season of sampling, and the type (source) of samples. Confirmation of ESBL production was conducted using double-disk diffusion methods following Clinical and Laboratory Standards Institute (CLSI) guidelines [43]. Confirmed ESBL *E. coli* isolates had a zone of inhibition of ≥5 mm for either Cefotaxime or Ceftazidime with Clavulanic acid compared to without Clavulanic acid. The isolates’ antimicrobial susceptibility was determined by broth microdilution methods using the NARMS Sensititre 14 antimicrobial drug panel. Data interpretation and categorization into susceptible, intermediate, and resistant were determined based on resistance breakpoints recommended by the CLSI of the U.S. [44,45], except for Streptomycin, which was determined based on resistance breakpoints recommended by the NARMS [46]. The number and percent resistance of ESBL *E. coli* isolates for the fourteen antimicrobials in the NARMS Sensititre panel are presented in Table 1.

### 4.2. Whole-Genome Sequencing

The template DNA for whole-genome sequencing (WGS) was extracted from an overnight culture of all selected *E. coli* isolates using the Qiagen DNeasy PowerLyser Microbial Kit following the manufacturer’s protocol. The purified DNA was quantified using a NanoDrop 2000 Spectrophotometer (Thermo Scientific, USA). The sequencing DNA library was prepared using the Nextera DNA Flex Library preparation kit (Illumina, San Diego, CA, USA) as previously described [47]. A Qubit 3.0 Fluorometer (ThermoFisher Scientific, Waltham, MA, USA) was used to quantify the library prep. WGS was performed on Illumina MiSeq with 300 bp paired-end reads. The average number of assembled contigs per sample was 96 (range 40 to 254), the average N50 was 201 kb (range 79 kb to 672 kb), and the total assembly length was 4.6 to 5.6 megabases (Mb).

Sequences were assembled using SPAdes 3.14.1 [48] and annotated with PROKKA [49] at default settings. The quality of genome assembly was assessed using Quast [50]. AMR genes, plasmids, and virulence genes were identified by the ABRicate pipeline, as previously described [51]. ABRicate included multiple databases including NCBI, CARD, ARG-ANNOT, ResFinder, MEGARES, EcOH, PlasmidFinder, Ecoli_VF, and VFDB. Reported AMR genes and plasmids were primarily based on summary results from ResFinder [52] and PlasmidFinder [53] databases of ABRicate program, respectively. The NCBI’s AMRfinderPlus database (version 3.10.5, Bethesda, MD, USA) [54] was used for the detection of AMR-associated point mutations. A gene was considered present in the assembled genome of an isolate when there was 90% nucleotide identity and 80% coverage of length match with the specific gene in the database. In silico serotyping of the *E. coli* isolates was carried out using the EcOH database [55] in the ABRicate program, whereas *E. coli* isolates were phylogrouped using ClermonTyping [56], which divides them into seven main phylogroups termed A, B1, B2, C, D, E, and F.

### 4.3. Phylogenetic Analysis

Prokka (version 1.14.6) was used to annotate isolate genomes [49], and pan-genome analyses were conducted using Roary (version 3.13.0) with a minimum percentage identity for blastp of 95% [57]. Within Roary, MAFFT [58] was used to create a core genome alignment of genes present in 99% of the isolates. The core genome alignment was used to generate a phylogenetic tree on RaxMLGUI2.0 (RaxML—NG version 1.0.1) [59]. The best-fitting model identified was general time-reversible substitution with a Gamma rate of heterogeneity and a proportion of invariable sites estimate (GTR + I + G) and used to generate the maximum-likelihood phylogenetic tree with 500 bootstrap replicates. The phylogenetic tree was visualized and annotated using iTOL version 6.3 (https://itol.embl.de/itol.cgi; accessed on 19 July 2021) [60].

### 4.4. Statistical Analyses

The frequency of detection of AMR genes in ESBL *E. coli* from sheep and the abattoir environment was estimated. Parameters of central tendency and dispersion, bar diagrams, contingency tables, and simple proportions were obtained. The statistical significance was set at the alpha value of ≤ 0.05. Statistical analyses were performed using SAS version 9.4 (SAS Institute Inc., Cary, NC, USA).

## Figures and Tables

**Table 1:** Comparison of the number of resistant ESBL *E. coli* isolates (n = 113) that displayed genotypic and phenotypic resistance to antimicrobials.

Classes of Antimicrobials	Tested Drugs	Resistance Break Point ** (µg/mL)	Number of Isolates Resistant (%) ***	Phenotype: Resistant		Phenotype: Susceptible *	
				Genotype: Resistant	Genotype: Susceptible	Genotype: Resistant	Genotype: Susceptible
Beta–lactam combination agents	AUG2	≥32/16	9 (8.0)	7	2	4	100
Penicillins	AMP	≥32	113 (100.0)	113	0	0	0
Macrolides	AZI	≥32	45 (39.8)	40	5	15	53
Cephems	FOX	≥32	9 (8.0)	7	2	4	100
	XNL	≥8	112 (99.1)	112	0	1	0
	AXO	≥4	113 (100.0)	113	0	0	0
Phenicols	CHL	≥32	87 (77.0)	83	4	0	26
Quinolones	CIP	≥1	19 (16.8%)	19	0	50	44
	NAL	≥32	26 (23.0)	24	2	45	42
Aminoglycosides	GEN	≥16	21 (18.6)	21	0	67	25
	STR **	≥32	85 (75.2)	84	1	4	24
Tetracyclines	TET	≥16	110 (97.3)	103	7	1	2
Folate pathway antagonists	FIS	≥512	93 (82.3)	93	0	1	19
	SXT	≥4/76	40 (35.4)	38	2	4	69
Total				857	25	196	504

AUG2 = Amoxicillin/Clavulanic acid; AMP = Ampicillin; AZI = Azithromycin; FOX = Cefoxitin; XNL = Ceftiofur; AXO = Ceftriaxone; CHL = Chloramphenicol; CIP = Ciprofloxacin; NAL = Nalidixic Acid; GEN = Gentamicin; STR = Streptomycin; TET = Tetracycline; FIS = Sulfisoxazole; SXT = Trimethoprim/Sulfamethoxazole. MIC = Minimum inhibitory concentration; * For estimation of comparison parameters, the number of susceptible isolates included those with susceptible and intermediate MIC values; ** Resistance break points for Streptomycin were based on the National Antimicrobial Resistance Monitoring System (NARMS)-established breakpoints for antimicrobial resistance. ^***^ Number of isolates indicates number of phenotypically resistant isolates to the antimicrobial and percentage indicates proportion of isolates resistant to the antimicrobial among tested isolates. Total indicates the number of tests with a specific outcome.

**Table 2:** Number and percentage of beta-lactamase genes in ESBL *E. coli* isolates (n = 113) from sheep and abattoir environment and number of isolates carrying these genes among sample types and seasons.

Profile of Beta-Lactamase Genes	No. (%)	Sheep Samples (N = 65)				Environmental Samples (N = 48)				Seasons			
		CS	CC	SF	RAF	SS	LS	FS	WS	SP	SU	FA	WI
		10	20	28	7	10	21	8	9	44	27	15	27
*bla*_CTX-M-1_, *bla*_TEM-1A_	24 (21.2)	4	5	10	1	-	1	1	2	14	9	1	-
*bla*_CTX-M-32_, *bla*_CARB-2_	15 (13.3)	1	3	3	-	-	5	2	1	10	2	-	3
*bla*_CTX-M-32_	13 (11.5)	-	1	7	2	1	-	2	-	5	2	2	4
*bla*_CTX-M-15_	10 (8.8)	-	3	1	-	-	5	-	1	9	-	-	1
*bla*_CTX-M-55_	10 (8.8)	2	1	1	-	2	3	1	-	-	1	2	7
*bla*_CTX-M-65_	7 (6.2)	1	-	2	1	2	-	-	1	-	6	-	1
*bla*_CTX-M-1_, *bla*_CMY-2_, *bla*_TEM-1A_	6 (5.3)	-	1	1	1	-	1	1	1	-	-	2	4
*bla*_CTX-M-55_, *bla*_TEM-1B_	5 (4.4)	1	1	2	-	1	-	-	-	-	4	-	1
*bla*_CMY-2_, *bla*_TEM-1C_	4 (3.5)	-	-	-	-	-	4	-	-	4	-	-	-
*bla*_CTX-M-65_, bla_TEM-1A_	4 (3.5)	-	1	1	1	1	-	-	-	-	-	4	-
*bla*_CTX-M-65_, *bla*_TEM-1B_	3 (2.7)	1	-	-	1	1	-	-	-	1	-	-	2
*bla*_CTX-M-15_, *bla*_TEM-1B_	2 (1.8)	-	-	-	-	2	-	-	-	-	-	1	1
*bla*_CMY-2_	1 (0.9)	-	-	-	-	-	1	-	-	-	1	-	-
*bla*_CTX-M-1_	1 (0.9)	-	1	-	-	-	-	-	-	1	-	-	-
*bla*_CTX-M-1_, *bla*_TEM-1B_	1 (0.9)	-	1	-	-	-	-	-	-	-	1	-	-
*bla*_CTX-M-14_	1 (0.9)	-	-	-	-	-	-	-	1	-	1	-	-
*bla*_CTX-M-14_, *bla*_TEM-1B_	1 (0.9)	-	1	-	-	-	-	-	-	-	-	1	-
*bla*_CTX-M-15_, *bla*_TEM-1C_	1 (0.9)	-	-	-	-	-	-	-	1	-	-	-	1
*bla*_CTX-M-27_	1 (0.9)	-	-	-	-	-	-	1	-	-	-	-	1
*bla*_CTX-M-27_, *bla*_CARB-2_	1 (0.9)	-	-	-	-	-	-	-	1	-	-	1	-
*bla*_CTX-M-27_, *bla*_TEM-1B_	1 (0.9)	-	-	-	-	-	1	-	-	-	-	1	-
*bla*_CTX-M-32_, *bla*_TEM-1B_	1 (0.9)	-	1	-	-	-	-	-	-	-	-	-	1

CC = Carcass swabs, CS = Cecal content, SF = Sheep feces, RAF = Resting area feces, SS = Soil sample, LS = Lairage swab, FS = Feed sample, WS = Water sample, SP = Spring, SU = Summer, FA = Fall, WI = Winter

## Data Availability

Raw Illumina WGS reads were submitted to the National Center for Biotechnology Information (NCBI) database and all data can be found under BioProject accession number PRJNA293225.

## Supplementary Materials

The following are available online at https://www.mdpi.com/article/10.3390/pathogens10111480/s1, Table S1: Phenotypic AMR profiles, AMR genes, and AMR associated point mutations detected in ESBL *E. coli* isolates (n = 113) from sheep and abattoir environment, Table S2: Frequency of AMR determinants detected in ESBL *E. coli* isolates (n = 113) among sample sources and seasons, Table S3: Number and percentage of AMR genes other than beta-lactamases in ESBL *E. coli* isolates (n = 113) from sheep and abattoir environment. Table S4: Sampling methodology
