# Supplementary material for: Identification of CTX-M Type ESBL E. coli from Sheep and Their Abattoir Environment Using Whole-Genome Sequencing
Source: Pathogens. 2021 Nov 14;10(11):1480. doi: 10.3390/pathogens10111480 (PMC8618867; doi:10.3390/pathogens10111480)
Supplement: Supplementary file 1 [file pathogens-10-01480-s001.zip › pathogens-1411879-supplementary.pdf]

Table S1. Phenotypic AMR profiles, AMR genes, and AMR associated point mutations detected in ESBL *E. coli* isolates (n = 113) from sheep and abattoir environment.

Phenotypic antimicrobial resistance profile	Genotypic profiles	Serotypes	Isolates ID number
AMP-AXO-TET-XNL	$bla_{\text{CTX-M-15}}$, *qnrS1*, *tet(A)*	O10:H25	015, 021, 027, 030, 060, 063, 066, 081, 090
AMP-AXO-AZI-CHL-FIS-STR-SXT-TET-XNL	*aadA2*, $bla_{\text{CARB-2}}$, $bla_{\text{CTX-M-32}}$, *dfrA1*, *floR*, *mph(A)*, *qnrA1*, *sul1*, *tet(A)*	O8:H20	241, 296, 318, 476, 929, 1137, 1145, 1343
AMP-AXO-AZI-CHL-FIS-STR-TET-XNL	*aph(3'')-Ib*, *aph(6)-Id*, $bla_{\text{CTX-M-1}}$, $bla_{\text{TEM-1A}}$, *floR*, *mph(A)*, *qnrB19*, *sul2*, *tet(A)*	-:H32, O9:H30	102, 106, 117, 121, 144, 230, 376, 382
AMP-AXO-AZI-CHL-FIS-SXT-TET-XNL	$bla_{\text{CTX-M-32}}$, *dfrA1*, *floR*, *mph(A)*, *sul1*, *tet(A)*	-:H34, O8:H20	001, 007, 093, 343, 1066, 1075
AMP-AXO-CHL-FIS-STR-TET-XNL	*aph(3'')-Ib*, *aph(6)-Id*, $bla_{\text{CTX-M-1}}$, $bla_{\text{TEM-1A}}$, *floR*, *mph(A)*, *qnrB19*, *sul2*, *tet(A)*	-:H32, O27:H9	173, 182, 470, 494, 529
AMP-AXO-CHL-CIP-FIS-NAL-STR-TET-XNL	*aadA5*, *aph(3'')-Ib*, *aph(6)-Id*, $bla_{\text{CTX-M-55}}$, *dfrA17*, *floR*, *sul2*, *tet(A)*, *gyrA*_D87N, *gyrA*_S83L, *parC*_S80I	-:H23	1256, 1340, 1351, 1417
AMP-AXO-CHL-FIS-STR-TET-XNL	*aph(3'')-Ib*, *aph(6)-Id*, $bla_{\text{CTX-M-65}}$, $bla_{\text{TEM-1A}}$, *floR*, *sul2*, *tet(A)*	O8:H19, O178:H7	836, 866, 920, 963
AMP-AXO-FIS-GEN-STR-TET-XNL	*aac(3)-IId*, *aadA17*, $bla_{\text{CTX-M-65}}$, *lnu(F)*, *sul2*, *tet(A)*, *tet(C)*	-:H28	678, 722, 741, 801
AMP-AXO-TET-XNL	$bla_{\text{CMY-2}}$, $bla_{\text{TEM-1C}}$, *tet(A)*	O9:H30	069, 072, 075, 078
AMP-AUG2-AXO-AZI-CHL-FIS-FOX-STR-TET-XNL	*aph(3'')-Ib*, *aph(6)-Id*, $bla_{\text{CMY-2}}$, $bla_{\text{CTX-M-1}}$, $bla_{\text{TEM-1A}}$, *floR*, *mph(A)*, *sul2*, *tet(A)*	O100:H32	1128, 1222, 1319
AMP-AUG2-AXO-CHL-FIS-FOX-STR-TET-XNL	*aph(3'')-Ib*, *aph(6)-Id*, $bla_{\text{CMY-2}}$, $bla_{\text{CTX-M-1}}$, $bla_{\text{TEM-1A}}$, *floR*, *mph(A)*, *sul2*, *tet(A)*	O100:H32	1074, 1240, 1372
AMP-AXO-AZI-CHL-FIS-STR-TET-XNL	*aadA2*, $bla_{\text{CARB-2}}$, $bla_{\text{CTX-M-32}}$, *dfrA1*, *floR*, *mph(A)*, *qnrA1*, *sul1*, *tet(A)*, *tet(B)*	O7:H7	099, 142, 199
AMP-AXO-CHL-CIP-FIS-GEN-NAL-STR-SXT-TET-XNL	*aac(3)-IIa*, *aadA22*, *aph(3'')-Ib*, *aph(3')-Ia*, *aph(6)-Id*, $bla_{\text{CTX-M-55}}$, $bla_{\text{TEM-1B}}$, *dfrA14*, *floR*, *lnu(F)*, *sul2*, *sul3*, *tet(A)*, *gyrA*_D87N, *gyrA*_S83L, *parC_S80I*, *parE*_S458A	O9:H30	528, 552, 581
AMP-AXO-CHL-CIP-FIS-NAL-STR-TET-XNL	*aadA5*, *aph(3'')-Ib*, *aph(6)-Id*, $bla_{\text{CTX-M-55}}$, *dfrA17*, *sul2*, *tet(A)*, *gyrA*_D87N, *gyrA_S83L*, *parC_S80I*	-:H23	1310, 1346, 1360
AMP-AXO-AZI-CHL-FIS-NAL-STR-TET-XNL	*aph(3'')-Ib*, *aph(6)-Id*, $bla_{\text{CTX-M-1}}$, $bla_{\text{TEM-1A}}$, *floR*, *mph(A)*, *qnrB19*, *sul2*, *tet(A)*	-:H32	108, 125
AMP-AXO-AZI-CHL-FIS-STR-TET-XNL	*aph(3'')-Ib*, *aph(6)-Id*, $bla_{\text{CTX-M-1}}$, $bla_{\text{TEM-1A}}$, *floR*, *qnrB19*, *sul2*, *tet(A)*	-:H32	252, 328
AMP-AXO-CHL-CIP-FIS-GEN-NAL-STR-SXT-TET-XNL	*aac(3)-IId*, *aadA1*, *aadA2*, *aph(3')-Ia*, $bla_{\text{CTX-M-15}}$, $bla_{\text{TEM-1B}}$, *cmlA1*, *dfrA12*, *floR*, *lnu(F)*, *sul2*, *sul3*, *tet(A)*, *gyrA*_D87N, *gyrA*_S83L, *parC*_S80I, *parE*_S458A	O8:H9	1045, 1181
AMP-AXO-CHL-FIS-STR-TET-XNL	*aph(3'')-Ib*, *aph(6)-Id*, $bla_{\text{CTX-M-1}}$, $bla_{\text{TEM-1A}}$, *floR*, *qnrB19*, *sul2*, *tet(A)*	O9:H30	361, 425
AMP-AXO-FIS-GEN-STR-TET-XNL	*aac(3)-IIa*, *aadA22*, $bla_{\text{CTX-M-55}}$, *lnu(F)*, *sul3*, *tet(A)*	-:H45	1077, 1116
AMP-AUG2-AXO-AZI-CHL-FIS-FOX-GEN-STR-SXT-TET-XNL	*aadA2*, *aph(3'')-Ib*, $bla_{\text{CARB-2}}$, $bla_{\text{CTX-M-32}}$, *dfrA1*, *floR*, *qnrA1*, *sul1*	O8:H20	220
AMP-AUG2-AXO-AZI-CHL-FIS-FOX-NAL-STR-SXT-TET-XNL	*aph(3'')-Ib*, *aph(6)-Id*, $bla_{\text{CTX-M-1}}$, $bla_{\text{TEM-1A}}$, *floR*, *mph(A)*, *sul2*, *tet(A)*	-:H26	203
AMP-AUG2-AXO-CHL-FIS-FOX-STR-SXT-TET	*aadA7*, $bla_{\text{CMY-2}}$, *sul1*, *cyaA*_S352T	O25 or O31:H15	629
AMP-AXO-AZI-CHL-CIP-FIS-GEN-NAL-STR-SXT-TET-XNL	*aac(3)-IIa*, *aadA22*, *aadA5*, *aph(3')-Ia*, $bla_{\text{CTX-M-55}}$, $bla_{\text{TEM-1B}}$, *catA1*, *dfrA14*, *dfrA17*, *floR*, *lnu(F)*, *mph(A)*, *sul1*, *sul3*, *tet(B)*, *gyrA*_D87N, *gyrA*_S83L, *parC*_A56T, *parC*_S80I	O32:H10	522
AMP-AXO-AZI-CHL-CIP-FIS-NAL-STR-SXT-TET-XNL	*aadA1*, *aadA2*, *aph(3'')-Ib*, *aph(3')-Ia*, *aph(6)-Id*, $bla_{\text{CTX-M-27}}$, *cmlA1*, *dfrA12*, *erm(B)*, *floR*, *mph(A)*, *sul2*, *sul3*, *tet(A)*, *gyrA*_D87N, *gyrA*_S83L, *parC*_S80R, *parE*_L416F	O8 or O153:H32	1203
AMP-AXO-AZI-CHL-CIP-FIS-NAL-STR-SXT-TET-XNL	*aadA22*, *aadA5*, *aph(3'')-Ib*, *aph(3')-Ia*, *aph(6)-Id*, $bla_{\text{CTX-M-55}}$, $bla_{\text{TEM-1B}}$, *catA1*, *dfrA17*, *floR*, *lnu(F)*, *mph(A)*, *sul1*, *sul2*, *sul3*, *tet(B)*, *gyrA*_D87N, *gyrA*_S83L, *parC*_A56T, *parC*_S80I	O32:H10	1390

AMP-AXO-AZI-CHL-FIS-NAL-STR-TET-XNL	*aph(3'')-Ib*, *aph(6)-Id*, *bla*$_{CTX-M-1}$, *bla*$_{TEM-1A}$, *floR*, *sul2*, *tet(A)*	-:H26	24
AMP-AXO-AZI-CHL-FIS-STR-SXT-TET-XNL	*aadA2*, *bla*$_{CARB-2}$, *bla*$_{CTX-M-32}$, *dfrA1*, *floR*, *mph(A)*, *qnrA1*, *sul1*, *tet(A)*, *cyaA*_S352T, *uhpT*_E350Q	O17 or O77 or O106:H45	132
AMP-AXO-AZI-CHL-FIS-STR-SXT-TET-XNL	*aadA22*, *bla*$_{CARB-2}$, *bla*$_{CTX-M-32}$, *dfrA1*, *floR*, *lnu(F)*, *mph(A)*, *qnrA1*, *sul1*, *tet(A)*	O8:H20	278
AMP-AXO-AZI-CHL-FIS-STR-SXT-TET-XNL	*aadA5*, *aph(3'')-Ib*, *aph(6)-Id*, *bla*$_{CTX-M-55}$, *dfrA17*, *sul2*, *tet(A)*, *qnrA1*	O8:H20	452
AMP-AXO-AZI-CHL-FIS-STR-SXT-TET-XNL	*ant(3'')-Ia*, *bla*$_{CTX-M-32}$, *dfrA1*, *floR*, *mph(A)*, *qnrB19*, *sul1*, *tet(A)*	O45:H25	1212
AMP-AXO-AZI-CHL-FIS-SXT-TET-XNL	*bla*$_{CTX-M-32}$, *dfrA1*, *floR*, *sul1*, *tet(A)*	-:H21	1141
AMP-AXO-AZI-CHL-TET-XNL	*bla*$_{CTX-M-32}$, *dfrA1*, *floR*, *mph(A)*, *sul1*, *tet(A)*	O5:H21	1178
AMP-AXO-AZI-FIS-TET-XNL	*bla*$_{CTX-M-32}$, *mph(A)*, *qnrS1*, *sul1*, *tet(A)*	O8:H9	113
AMP-AXO-CHL-CIP-FIS-NAL-STR-SXT-TET-XNL	*aadA5*, *aph(3'')-Ib*, *aph(6)-Id*, *bla*$_{CTX-M-15}$, *bla*$_{TEM-1C}$, *catA1*, *dfrA17*, *mph(A)*, *sul1*, *sul2*, *tet(A)* , *gyrA*_D87N, *gyrA*_S83L, *parC*_A56T, *parC*_S80I	O22 or O32:H9	1325
AMP-AXO-CHL-CIP-FIS-NAL-STR-SXT-TET-XNL	*aadA5*, *aph(3'')-Ib*, *aph(6)-Id*, *bla*$_{CTX-M-15}$, *catA1*, *dfrA17*, *mph(A)*, *sul1*, *sul2*, *tet(A)*, *gyrA*_D87N, *gyrA*_S83L, *parC*_A56T, *parC*_S80I	O22 or O32:H9	1280
AMP-AXO-CHL-CIP-FIS-NAL-SXT-TET-XNL	*aadA5*, *aph(3'')-Ib*, *aph(6)-Id*, *bla*$_{CTX-M-55}$, *dfrA17*, *floR*, *tet(A)*, *gyrA*_D87N, *gyrA_S83L*, *parC*_S80I	O5:H20	750
AMP-AXO-CHL-CIP-NAL-TET-XNL	*aph(3')-IIa*, *bla*$_{CTX-M-32}$, *floR*, *tet(A)*, *gyrA*_D87N, *gyrA*_S83L, *parC*_S80I, *parE*_S458A	O74:H23	1344
AMP-AXO-CHL-FIS-GEN-NAL-STR-SXT-TET-XNL	*aac(3)-IId*, *aadA17*, *aph(3')-Ia*, *aph(6)-Id*, *bla*$_{CTX-M-65}$, *bla*$_{TEM-1B}$, *dfrA14*, *floR*, *lnu(F)*, *aph(6)-Id*, *sul2*, *sul3*, *tet(A)*, *tet(B)*, *gyrA*_S83L	O23:H25	1387
AMP-AXO-CHL-FIS-GEN-STR-SXT-TET-XNL	*aac(3)-IId*, *aadA2*, *aph(3'')-Ib*, *aph(6)-Id*, *bla*$_{CTX-M-65}$, *dfrA23*, *floR*, *sul1*, *sul2*, *tet(A)*	O29:H34	674
AMP-AXO-CHL-FIS-GEN-STR-SXT-TET-XNL	*aac(3)-IId*, *aadA5*, *aph(3'')-Ib*, *aph(6)-Id*, *bla*$_{CTX-M-1}$, *bla*$_{TEM-1A}$, *catA1*, *dfrA17*, *floR*, *mph(A)*, *sul2*, *tet(A)*, *tet(B)*	O9:H34	659
AMP-AXO-CHL-FIS-GEN-STR-SXT-TET-XNL	*aac(3)-VIa*, *aph(3'')-Ib*, *aph(3')-Ia*, *aph(6)-Id*, *bla*$_{CTX-M-14}$, *dfrA1*, *floR*, *qnrB19*, *sul1*, *sul2*, *tet(A)*	O13 or O129:H2	816
AMP-AXO-CHL-FIS-GEN-STR-SXT-XNL	*aac(3)-IId*, *aadA1*, *aadA2*, *aph(3')-Ia*, *bla*$_{CTX-M-1}$, *bla*$_{TEM-1B}$, *cmlA1*, *dfrA12*, *floR*, *lnu(F)*, *sul2*, *sul3*	O8:H19	708
AMP-AXO-CHL-FIS-NAL-STR-TET-XNL	*aph(3'')-Ib*, *aph(3')-Ia*, *aph(6)-Id*, *bla*$_{CTX-M-65}$, *bla*$_{TEM-1B}$, *floR*, *sul2*, *sul3*, *tet(A)*, *tet(M)*, *gyrA*_S83L	O87:H25	42
AMP-AXO-CHL-FIS-STR-SXT-TET-XNL	*aadA2*, *bla*$_{CARB-2}$, *bla*$_{CTX-M-32}$, *dfrA1*, *floR*, *mph(A)*, *qnrA1*, *sul1*, *tet(A)*	O8:H20	129
AMP-AXO-CHL-FIS-STR-TET-XNL	*aadA1*, *bla*$_{CTX-M-32}$, *bla*$_{TEM-1B}$, *floR*, *lnu(F)*, *qnrB19*, *sul3*, *tet(A)*, *uhpT_E350Q*	O53:H38	1228
AMP-AXO-CHL-FIS-STR-TET-XNL	*aph(3'')-Ib*, *aph(6)-Id*, *bla*$_{CTX-M-1}$, *bla*$_{TEM-1A}$, *floR*, *mph(A)*, *sul2*, *tet(A)*, *uhpT*_E350Q, *uhpT*_E350Q	O10:H29	955
AMP-AXO-CHL-FIS-STR-TET-XNL	*aph(3'')-Ib*, *aph(6)-Id*, *bla*$_{CTX-M-1}$, *bla*$_{TEM-1A}$, *floR*, *sul2*, *tet(A)*	-:H26	238
AMP-AXO-CHL-FIS-STR-TET-XNL	*aph(3'')-Ib*, *aph(6)-Id*, *bla*$_{CTX-M-32}$, *floR*, *sul2*, *tet(A)*	O62:H12	819
AMP-AXO-CHL-GEN-TET-XNL	*aac(3)-IId*, *aph(3')-IIa*, *bla*$_{CTX-M-65}$, *floR*, *tet(A)*	-:H26	407
AMP-AXO-FIS-GEN-STR-TET-XNL	*aac(3)-IId*, *aadA17*, *bla*$_{CTX-M-65}$, *bla*$_{TEM-1B}$, *lnu(F)*, *sul2*, *sul3*, *tet(A)*	O118 or O151:H12	1237
AMP-AXO-FIS-STR-SXT-TET-XNL	*ant(3'')-Ia*, *aph(3')-Ia*, *aph(6)-Id*, *bla*$_{CTX-M-14}$, *bla*$_{TEM-1B}$, *dfrA1*, *mph(B)*, *aph(6)-Id*, *sul1*, *sul2*, *tet(B)*, *cyaA*_S352T	O17 or O77:H7	851
AMP-AXO-GEN-TET-XNL	*aac(3)-IId*, *bla*$_{CTX-M-65}$, *tet(A)*, *uhpT*_E350Q	O159:H28	1414
AMP-AXO-STR-TET-XNL	*aph(6)-Id*, *bla*$_{CTX-M-27}$, *bla*$_{TEM-1B}$, *aph(6)-Id*, *tet(B)*	O23:H25	883
AMP-AXO-XNL	*bla*$_{CTX-M-1}$, *mph(A)*	-:H26	18
AMP-AXO-XNL	*bla*$_{CTX-M-32}$	O24:H32	267

AMP = Ampicillin, AUG2 = Amoxicillin/Clavulanic acid, AXO = Ceftriaxone, AZI = Azithromycin, CHL = Chloramphenicol, CIP = Ciprofloxacin, FIS = Sulfisoxazole, FOX = Cefoxitin, GEN = Gentamicin, NAL = Nalidixic Acid, STR = Streptomycin, SXT = Trimethoprim/Sulfamethoxazole, TET = Tetracycline and XNL = Ceftiofur.

Table S2. Frequency of AMR determinants detected in ESBL *E. coli* isolates (n=113) among sample sources and seasons.

Categories	Unique type genes, point mutations, and plasmids	Number of isolates (N=113)	Sample source		Seasons			
			Environment (n=48)	Sheep (N=65)	Spring (n=44)	Summer (n=27)	Fall (n=15)	Winter (n=27)
Aminoglycosides	*aph(3'')-Ib*	56	20	36	16	16	9	15
	aph(6)-Id	56	20	36	15	16	9	16
	aadA2	17	9	8	9	3	1	4
	aac(3)-IId	13	7	6	0	8	1	4
	aadA5	13	8	5	0	3	0	10
	aph(3')-Ia	13	6	7	1	6	2	4
	ant(3'')-Ia	12	5	7	0	5	2	5
	aac(3)-IIa	6	2	4	0	4	2	0
	aph(3')-IIa	2	1	1	0	1	0	1
	aac(3)-VIa	1	1	0	0	1	0	0
	aadA22	1	1	0	1	0	0	0
	aadA7	1	1	0	0	1	0	0
Beta-lactamase	*bla*TEM-1A	34	8	26	14	9	7	4
	*bla*CTX-M-1	32	7	25	15	10	3	4
	*bla*CTX-M-32	29	11	18	15	4	2	8
	*bla*CARB-2	16	9	7	10	2	1	3
	*bla*CTX-M-55	15	7	8	0	5	2	8
	*bla*CTX-M-65	14	5	9	1	6	4	3
	*bla*TEM-1B	14	5	9	1	5	3	5
	*bla*CTX-M-15	13	9	4	9	0	1	3
	*bla*CMY-2	11	8	3	4	1	2	4
	*bla*TEM-1C	5	5	0	4	0	0	1
	*bla*CTX-M-27	3	3	0	0	0	2	1
	*bla*CTX-M-14	2	1	1	0	1	1	0
Phenicols	*floR*	81	29	52	28	22	11	20
	catA1	5	4	1	0	2	0	3
	cmlA1	4	3	1	0	1	1	2
Folate pathway antagonists								
Trimethoprim	*dfrA1*	26	11	15	13	3	4	6
	dfrA17	6	5	1	0	3	0	3
	dfrA14	5	1	4	0	4	0	1
	dfrA12	4	3	1	0	1	1	2
	dfrA10	1	1	0	0	1	0	0
	dfrA23	1	0	1	0	1	0	0
Sulfonamides	*sul2*	61	20	41	15	19	9	18
	sul1	33	15	18	14	6	4	9
	sul3	15	7	8	1	5	3	6
Macrolides	*mph(A)*	54	20	34	25	10	6	13
	erm(B)	1	1	0	0	0	0	1
	mph(B)	1	0	1	0	0	1	0
Quinolones/Floroquinolones								
Genes	*qnrB19*	22	3	19	11	9	0	2
	qnrA1	16	9	7	10	2	1	3
	qnrS1	10	5	5	10	0	0	0
Point mutations	*gyrA*_S83L	21	11	10	1	5	1	14
	*gyrA*_D87N	19	10	9	0	5	1	13
	*parC*_S80I	18	9	9	0	5	1	12
	*parE*_S458A	6	2	4	0	3	1	2
	*parC*_A56T	4	3	1	0	1	0	3
	*parE*_L416F	1	1	0	0	0	0	1
	*parC*_S80R	1	1	0	0	0	0	1
Tetracyclines	*tet(A)*	99	43	56	41	22	10	26
	tet(B)	9	4	5	3	2	2	2
	tet(C)	4	1	3	0	4	0	0
	tet(M)	2	0	2	1	1	0	0
Lincosamides	*lnu(F)*	18	8	10	1	9	3	5
Fosfomycin	*uhpT*_E350Q	4	1	3	1	0	1	2
	*cyaA*_S352T	3	1	2	1	1	1	0
Plasmids	IncR	57	18	39	28	12	6	11
	IncFIB	34	19	15	13	9	7	5

Col440I	23	4	19	11	10	0	2
IncFII	18	12	6	14	0	3	1
IncX1	17	7	10	0	6	0	11
IncI1_Alpha	15	10	5	4	1	1	9
IncHI2	14	5	9	1	6	4	3
IncFIC	13	6	7	0	5	6	2
p0111	10	3	7	0	3	0	7
IncFIA	7	4	3	0	1	4	2
IncX4	6	3	3	0	0	2	4
IncFII(pCoo)	4	4	0	4	0	0	0
Col156	3	2	1	0	1	2	0
IncA/C2	2	2	0	1	1	0	0
Col(MG828)	1	0	1	0	1	0	0
ColRNAI	1	0	1	1	0	0	0
IncHI1A	1	1	0	0	1	0	0
IncHI1B(R27)	1	1	0	0	1	0	0
IncN	1	1	0	1	0	0	0

Table S3. Number and percentage of AMR genes other than beta-lactamases in ESBL *E. coli* isolates (n = 113) from sheep and abattoir environment.

Antimicrobial Class	Genotypic Mechanisms of Resistance	Total (%)	Sources of ESBL *E. coli* Isolates (Number of Isolates)							
			Sheep Samples (n = 65)				Environment Samples (n = 48)			
			CS (n = 10)	CC (n = 20)	SF (n = 28)	RAF (n = 7)	SS (n = 10)	LS (n = 21)	FS (n = 8)	WS (n = 9)
Aminoglycosides	*aph(3'')-Ib, aph(6)-Id*	35 (31.0)	4	7	12	3	1	3	2	3
	aadA2	14 (12.4)	1	3	3	-	-	3	2	2
	aadA5, aph(3'')-Ib, aph(6)-Id	9 (8.0)	2	1	1	-	1	3	-	1
	aac(3)-IId, ant(3'')-Ia	5 (4.4)	1	-	1	2	1	-	-	-
	aac(3)-IIa, aph(3')-Ia, aph(3'')-Ib, aph(6)-Id	3 (2.7)	1	1	1	-	-	-	-	-
	aac(3)-IId, ant(3'')-Ia, aph(3')-Ia	3 (2.7)	-	1	-	-	2	-	-	-
	aph(3')-Ia, aph(3'')-Ib, aph(6)-Id	2 (1.8)	1	-	-	-	-	-	1	-
	aac(3)-IIa	2 (1.8)	-	-	-	-	1	1	-	-
	aac(3)-IId, aadA2, aph(3'')-Ib, aph(6)-Id	1 (0.9)	-	-	1	-	-	-	-	-
	aac(3)-VIa, aph(3')-Ia, aph(3'')-Ib, aph(6)-Id	1 (0.9)	-	-	-	-	-	-	-	1
	aadA2, ant(3'')-Ia, aph(3')-Ia, aph(3'')-Ib, aph(6)-Id	1 (0.9)	-	-	-	-	-	-	1	-
	aadA5, aph(3')-Ia, aph(3'')-Ib, aph(6)-Id	1 (0.9)	-	-	-	-	1	-	-	-
	ant(3'')-Ia, aph(3')-Ia, aph(3'')-Ib, aph(6)-Id	1 (0.9)	-	1	-	-	-	-	-	-
	aac(3)-IId, aadA5, aph(3'')-Ib, aph(6)-Id	1 (0.9)	-	-	-	-	-	-	1	-
	aac(3)-IId, ant(3'')-Ia, aph(3')-Ia, aph(6)-Id	1 (0.9)	-	-	-	-	1	-	-	-
	aac(3)-IIa, aadA5, aph(3')-Ia	1 (0.9)	-	-	1	-	-	-	-	-
	aadA2, aph(3'')-Ib	1 (0.9)	-	-	-	-	-	1	-	-
	aac(3)-IId, aph(3')-IIa	1 (0.9)	-	-	-	-	1	-	-	-
	aac(3)-IId	1 (0.9)	-	-	-	-	-	-	-	1
	aadA7	1 (0.9)	-	-	-	-	-	1	-	-
	aadA22	1 (0.9)	-	-	-	-	-	1	-	-
	ant(3'')-Ia	1 (0.9)	-	-	1	-	-	-	-	-
	aph(3')-IIa	1 (0.9)	-	-	1	-	-	-	-	-
Tetracyclines	*tet(A)*	89 (78.8)	7	14	23	5	6	18	7	9
	tet(A), tet(B)	5 (4.4)	1	1	1	-	1	-	1	-
	tet(B)	4 (3.5)	-	1	1	-	1	1	-	-
	tet(A), tet(C)	4 (3.5)	1	-	1	1	1	-	-	-
	tet(A), tet(M)	1 (0.9)	1	-	-	-	-	-	-	-
	tet(M)	1 (0.9)	-	1	-	-	-	-	-	-
Folate pathway antagonists	*sul2*	45 (39.8)	7	8	14	4	3	3	3	3
	dfrA1, sul1	25 (22.1)	1	3	8	2	1	5	3	2
	dfrA14, sul2, sul3	4 (3.5)	1	1	1	-	1	-	-	-

	dfrA12, sul2, sul3	4 (3.5)	-	1	-	-	2	-	1	-
	sul3	3 (2.7)	-	1	-	-	1	1	-	-
	dfrA17, sul1, sul2	2 (1.8)	-	-	-	-	-	1	-	1
	dfrA17	2 (1.8)	-	-	-	-	-	1	1	-
	sul2, sul3	2 (1.8)	1	-	-	1	-	-	-	-
	sul1	2 (1.8)	-	-	1	-	-	1	-	-
	dfrA17, sul1, sul2, sul3	1 (0.9)	-	-	-	-	1	-	-	-
	dfrA14, dfrA17, sul1, sul3	1 (0.9)	-	-	1	-	-	-	-	-
	dfrA23, sul1, sul2	1 (0.9)	-	-	1	-	-	-	-	-
	dfrA10, sul2	1 (0.9)	-	-	-	-	-	-	-	1
	dfrA1	1 (0.9)	-	1	-	-	-	-	-	-
Phenicols	*floR*	74 (65.5)	9	12	24	5	4	8	6	6
	floR, cmlA1	4 (3.5)	-	1	-	-	2	-	1	-
	floR, CatA1	3 (2.7)	-	-	1	-	1	-	1	-
	CatA1	2 (1.8)	-	-	-	-	-	1	-	1
Macrolides	*mph(A)*	53 (46.9)	4	8	19	3	2	7	5	5
	mph(B)	1 (0.9)	-	1	-	-	-	-	-	-
	Inu(F)	1 (0.9)	1	-	-	-	-	-	-	-
	erm(B), mph(A)	1 (0.9)	-	-	-	-	-	-	1	-
Quinolones	*qnrB19*	22 (19.5)	3	5	10	1	-	1	-	2
	qnrA1	16 (14.2)	1	3	3	-	-	5	2	2
	qnrS1	10 (8.8)	-	3	2	-	-	4	-	1
	*gyrA*_D87N, *gyrA*_S83L, *parC*_S80I	8 (7.1)	2	1	1	-	1	2	1	-
	*gyrA*_D87N, *gyrA_S83L*, *parC*_S80I, *parE*_S458A	6 (5.3)	1	1	2	-	2	-	-	-
	*gyrA*_D87N, *gyrA*_S83L, *parC*_A56T, *parC*_S80I	4 (3.5)	-	-	1	-	1	1	-	1
	*gyrA*_S83L	2 (1.8)	1	-	-	-	1	-	-	-
	*gyrA*_D87N, *gyrA*_S83L, *parC*_S80R, *parE*_L416F	1 (0.9)	-	-	-	-	-	-	1	-
Fosfomycin	*uhpT*_E350Q	3 (2.7)	-	1	1	-	-	-	-	1
	*cyaA*_S352T	2 (1.8)	-	1	-	-	-	1	-	-
	*cyaA*_S352T, *uhpT*_E350Q	1 (0.9)	-	1	-	-	-	-	-	-
Lincosamides	*Inu(F)*	18 (15.9)	2	3	3	2	6	2	-	-

CC = Carcass swabs, CS= Cecal content, SF= -Sheep feces, RAF= Resting area feces, SS= Soil sample, LS= Lairage swab, FS= Feed sample, WS= Water sample

Table S4. Sampling methodology.

Sources of Sample	Type of Sheep Samples	Samples Collected Per Visit	Number of Samples Collected	Sampling Method*	Amount (Approximate)	Sampling Material
Sheep samples (n = 780)	Sheep feces	13–20	220	Directly from rectum	5 grams	Sterile whirl-pack bags (Nasco™)
	Cecal contents	15–20	224	Milked from the cecum	5 grams	Sterile screw-topped cups
	Carcass swabs	16–30	246	Sponging at flank, brisket, and rump (approximately 100 cm^2 area per site) of each carcass	One swab/ carcass	Sterile sponges (Nasco™) presoaked with 10 ml of BPW (Difco™)
Abattoir environment samples (n = 348)	Abattoir resting area feces	10	90	Freshly dropped sheep feces from the ground	5 grams	Sterile whirl-pack bags (Nasco™)
	Lairage swab	10	120	Swabbing the floor of occupied pens	Five swabs per pen	Sterile sponges (Nasco™) presoaked with 10 ml of BPW (Difco™)
	Soil samples	10	90	10 grams of samples from different locations	10 grams	Sterile Whirl-pack bags (Nasco™)
	Animal feed	5–6	69	10 grams of samples	10 grams	Sterile screw-topped cups
	Animal drinking water	5–6	69		10ml	Sterile screw-topped cups

*Samples collected aseptically using separate sterile gloves. Sheep feces, cecal contents, and carcass swabs were collected from the same animal immediately after evisceration. Resting area feces and soil samples were not collected during the first three months.
